# Supplementary material for: Occurrence of Antimicrobial-Resistant Bacteria in Intestinal Contents of Wild Marine Fish in Chile
Source: Antibiotics (Basel). 2024 Apr 5;13(4):332. doi: 10.3390/antibiotics13040332 (PMC11047320; doi:10.3390/antibiotics13040332)
Supplement: Supplementary file 1 [file antibiotics-13-00332-s001.zip › antibiotics-2939038-supplementary/tables/Table S1.pdf]

Table S1. Identification and source of resistant isolates from intestinal content of pelagic fishes.

Isolate	Fish species	Accession N°	Closest species (Accession N°)	% Identity
NCIA21	*Seriola lalandi*	PP277037	*Shewanella colwelliana* (AY653177)	98.79
NCIA22	*Seriola lalandi*	PP277038	*Vibrio* sp. (KM369839)	97.40
NCIA23	*Seriola lalandi*	PP294768	*Vibrio kanaoae* (AJ316193)	99.68
NCIA24	*Seriola lalandi*	PP294769	*Vibrio lentus* (AY292935)	100
NCIA25	*Seriola lalandi*	PP294770	*Pseudomonas* sp. (JQ012958)	99.10
NCIA26	*Seriola lalandi*	PP294771	*Pseudomonas* sp. (EF451704)	98.60
NCIA27	*Seriola lalandi*	PP263371	*Shewanella algidipiscicola* (NR_041297)	99.14
NCIA28	*Seriola lalandi*	PP264675	*Pseudomonas crudilactis* (NR_179985)	99.46
NCIA29	*Seriola lalandi*	PP296421	*Photobacterium* sp. (EU090138)	98.00
NCIO14	*Seriola lalandi*	PP264921	*Psychrobacter submarinus* (NR_027225)	100
NCIO15	*Seriola lalandi*	PP264956	*Psychrobacter piscatorii* (NR_112807)	98.13
NCIO16	*Seriola lalandi*	PP265062	*Psychrobacter piscatorii* (NR_112807)	100
NCIO17	*Seriola lalandi*	PP265078	*Psychrobacter maritimus* (NR_027225)	100
NCIO18	*Seriola lalandi*	PP296423	*Moellerella wisconsensis* (GQ451444)	99.10
NCIO19	*Seriola lalandi*	PP297230	*Providencia alcalifaciens* (AB480755)	97.94
NCIS20	*Seriola lalandi*	PP265264	*Shewanella algidipiscicola* (NR_041297)	99.14
NCIS22	*Seriola lalandi*	PP301860	*Shewanella algidipiscicola* (NR_041297)	98.72
NCIS23	*Seriola lalandi*	PP297271	*Vibrio* sp. (FJ457601)	100
NCIS24	*Seriola lalandi*	PP265273	*Vibrio toranzoniae* (NR_117680)	99.25
NCIS25	*Seriola lalandi*	PP297450	*Pseudomonas gessardii* (KF478209)	99.30
NCIS26	*Seriola lalandi*	PP297455	*Pseudomonas fulva* (AB047001)	83.40
NCIA56	*Scomber japonicus*	PP297464	*Vibrio kanaloae* (JN128262)	98.20
NCIA57	*Scomber japonicus*	PP301828	*Pseudomonas jessenii* (AM933510)	100
NCIA58	*Scomber japonicus*	PP301894	*Pseudomonas* sp. (HQ224649)	99.40
NCIA59	*Scomber japonicus*	PP265277	*Pseudomonas crudilactis* (NR_179985)	99.57
NCIO36	*Scomber japonicus*	PP301968	*Vibrio* sp. (AB038029)	98.10
NCIO37	*Scomber japonicus*	PP301969	*Pseudomonas* sp. (KT923307)	98.50
NCIO38	*Scomber japonicus*	PP301988	*Shewanella arctica* (R_178284)	97.83
NCIO39	*Scomber japonicus*	PP302050	*Pseudomonas fluorescens* (AB204715)	100
NCIS45	*Scomber japonicus*	PP265278	*Brochothrix thermosphacta* (NR_113587)	99.88
NCIS46	*Scomber japonicus*	PP309615	*Pseudomonas* sp. (KJ152096)	99.30
NCIS47	*Scomber japonicus*	PP265285	*Pseudomonas deceptione* (NR_117552)	99.89
NCIA1	*Sarda chiliensis*	PP309808	*Pseudomonas* sp. (FJ002582)	100
NCIA2	*Sarda chiliensis*	PP309816	*Pseudomonas fluorescens* (AF228367)	99.86
NCIA3	*Sarda chiliensis*	PP309838	*Pseudomonas mandelii* (AY179326)	99.86
NCIA4	*Sarda chiliensis*	PP309853	*Pseudomonas* sp. (AY014829)	99.79

NCIA5	*Sarda chiliensis*	PP309854	*Pseudomonas* sp. (FJ002582)	99.93
NCIF4	*Sarda chiliensis*	PP309856	*Pseudomonas* sp. (FJ002582)	99.93
NCIF5	*Sarda chiliensis*	PP309920	*Pseudomonas* sp. (AJ002801)	99.13
NCIF6	*Sarda chiliensis*	PP309919	*Pseudomonas* sp. (AY303304)	98.40
NCIF7	*Sarda chiliensis*	PP266000	*Pseudomonas lactucae* (NR_179365)	99.46
NCIF8	*Sarda chiliensis*	PP266006	*Pseudomonas crudilactis* (OR673555)	99.46
NCIF9	*Sarda chiliensis*	PP317017	*Pseudomonas* sp. (AJ002801)	99.21
NCIO2	*Sarda chiliensis*	PP317016	*Acinetobacter johnsonii* (FJ976603)	100
NCIO4	*Sarda chiliensis*	PP272032	*Brochothrix thermosphacta* (NR_113587)	99.88
NCIO5	*Sarda chiliensis*	PP266007	*Brochothrix thermosphacta* (NR_113587)	99.65
NCIO7	*Sarda chiliensis*	PP317133	*Shewanella baltica* (AY771739)	96.10
NCIO8	*Sarda chiliensis*	PP317291	*Pseudomonas fragi* (AM062695)	99.86
NCIO9	*Sarda chiliensis*	PP317292	*Pseudomonas fragi* (AM062695)	99.71
NCIO10	*Sarda chiliensis*	PP317367	*Pseudomonas fragi* (AM062695)	99.79
NCIS3	*Sarda chiliensis*	PP317368	*Shewanella* sp. (AF022401)	99.79
NCIS4	*Sarda chiliensis*	PP266434	*Brochothrix thermosphacta* (NR_113587)	100
NCIS5	*Sarda chiliensis*	PP270284	*Brochothrix thermosphacta* (NR_113587)	98.93
NCIS6	*Sarda chiliensis*	PP266932	*Pseudomonas frederiksber* (NR_117177)	99.36
NCIS7	*Sarda chiliensis*	PP266933	*Myroides phaeus* (NR_117475)	99.46
NCIS8	*Sarda chiliensis*	PP320223	*Pseudomonas fragi* (AM062695)	99.71
NCIA60	*Trachurus murphyi*	PP272033	*Pseudomonas crudilactis* (NR_179985)	99.46
NCIA61	*Trachurus murphyi*	PP320224	*Shewanella* sp. (FJ169982)	97.30
NCIA62	*Trachurus murphyi*	PP320225	*Pseudomonas* sp. (KR054995)	98.20
NCIF1	*Trachurus murphyi*	PP320227	*Pseudomonas fragi* (AF094733)	99.64
NCIF2	*Trachurus murphyi*	PP266935	*Pseudomonas kielensis* (NR_181570)	99.47
NCIF3	*Trachurus murphyi*	PP320226	*Brevibacterium* sp. (JQ680447)	99.30
NCIF24	*Trachurus murphyi*	PP266937	*Pseudomonas crudilactis* (NR_179985)	99.57
NCIF25	*Trachurus murphyi*	PP320229	*Pseudomonas* sp. (FJ379535)	100
NCIF26	*Trachurus murphyi*	PP320228	*Pseudomonas* sp. (AM421975)	99.20
NCIO1	*Trachurus murphyi*	PP325872	*Pseudomonas* sp. (GU062532)	99.86
NCIO40	*Trachurus murphyi*	PP325873	*Shewanella putrefaciens* (AB205573)	100
NCIO41	*Trachurus murphyi*	PP325876	*Pseudomonas* sp. (AM409370)	97.30
NCIO42	*Trachurus murphyi*	PP267450	*Psychrobacter immobilis* (NR_113805)	99.77
NCIO43	*Trachurus murphyi*	PP325875	*Shewanella putrefaciens* (AB205573)	99.40
NCIS1	*Trachurus murphyi*	PP325878	*Shewanella marina* (EU290154)	98.60
NCIS48	*Trachurus murphyi*	PP267452	*Brochothrix thermosphacta* (NR_113587)	99.46
NCIS49	*Trachurus murphyi*	PP267451	*Shewanella halifaxensis* (NR_074822)	99.77
NCIS50	*Trachurus murphyi*	PP325877	*Shewanella marina* (EU290154)	97.10
