# Supplementary material for: Occurrence of Antimicrobial-Resistant Bacteria in Intestinal Contents of Wild Marine Fish in Chile
Source: Antibiotics (Basel). 2024 Apr 5;13(4):332. doi: 10.3390/antibiotics13040332 (PMC11047320; doi:10.3390/antibiotics13040332)
Supplement: Supplementary file 1 [file antibiotics-13-00332-s001.zip › antibiotics-2939038-supplementary/tables/Table S2.pdf]

Table S2. Identification and source of resistant isolates from intestinal content of demersal fishes.

Isolate	Fish species	Accession Nº	Closest species (Accession Nº)	% Identity
NCIA6	*Merluccius gayi gayi*	PP326983	*Pseudomonas fluorescens* (AF228367)	85.50
NCIA7	*Merluccius gayi gayi*	PP326984	*Vibrio* sp. (DQ146980)	99.29
NCIA8	*Merluccius gayi gayi*	PP326985	*Pseudomonas fluorescens* (AF228367)	98.60
NCIA9	*Merluccius gayi gayi*	PP326986	*Pseudomonas* sp. (AY014829)	99.86
NCIA10	*Merluccius gayi gayi*	PP326988	*Pseudomonas* sp. (JQ012960)	100
NCIA11	*Merluccius gayi gayi*	PP267453	*Pseudomonas crudilactis* (NR_179985)	99.36
NCIF10	*Merluccius gayi gayi*	PP326990	*Pseudomonas* sp. (FJ002582)	99.79
NCIF12	*Merluccius gayi gayi*	PP326991	*Pseudomonas* sp. (FJ002582)	99.93
NCIF13	*Merluccius gayi gayi*	PP326992	*Pseudomonas* sp. (FJ379535)	97.40
NCIO11	*Merluccius gayi gayi*	PP326994	*Acinetobacter johnsonii* (EU977635)	99.84
NCIO12	*Merluccius gayi gayi*	PP267454	*Psychrobacter cibarius* (NR_043057)	99.36
NCIO13	*Merluccius gayi gayi*	PP272034	*Psychrobacter maritimus* (NR_027225)	100
NCIS9	*Merluccius gayi gayi*	PP326993	*Pseudomonas* sp. (DQ683573)	96.90
NCIS10	*Merluccius gayi gayi*	PP326995	*Pseudomonas* sp. (AM398216)	100
NCIS11	*Merluccius gayi gayi*	PP326996	*Vibrio toranzoniae* (HE978311)	100
NCIS12	*Merluccius gayi gayi*	PP326999	*Pseudomonas fluorescens* (AF228367)	100
NCIA46	*Menticirrhus ophicephalus*	PP326998	*Vibrio lentus* (AY292935)	99.50
NCIA47	*Menticirrhus ophicephalus*	PP327001	*Vibrio lentus* (AY292935)	99.10
NCIA48	*Menticirrhus ophicephalus*	PP327000	*Vibrio toranzoniae* (HE978311)	100
NCIA49	*Menticirrhus ophicephalus*	PP327004	*Pseudomonas* sp. (FJ379535)	100
NCIA50	*Menticirrhus ophicephalus*	PP327006	*Pseudomonas* sp. (JQ012960)	100
NCIA51	*Menticirrhus ophicephalus*	PP327007	*Shewanella* sp. (FJ231175)	100
NCIA52	*Menticirrhus ophicephalus*	PP327011	*Vibrio kanaloae* (NR_114804)	99.34
NCIA53	*Menticirrhus ophicephalus*	PP327010	*Photobacterium* sp. (FJ457575)	97.60
NCIA54	*Menticirrhus ophicephalus*	PP327012	*Vibrio lentus* (AY292935)	99.00
NCIA55	*Menticirrhus ophicephalus*	PP327013	*Vibrio toranzoniae* (HE978311)	100
NCIF19	*Menticirrhus ophicephalus*	PP327015	*Pseudomonas* sp (JQ012960)	100
NCIF20	*Menticirrhus ophicephalus*	PP327014	*Pseudomonas* sp. (HM196354)	98.60
NCIF21	*Menticirrhus ophicephalus*	PP327017	*Pseudomonas jessenii* (AM933510)	100
NCIF22	*Menticirrhus ophicephalus*	PP327016	*Pseudomonas gessardii* (AF074384)	98.10
NCIF23	*Menticirrhus ophicephalus*	PP327028	*Pseudomonas* sp. (AY573031)	99.30
NCIO26	*Menticirrhus ophicephalus*	PP327029	*Moellerella wisconsensis* (KP058388)	99.40
NCIO27	*Menticirrhus ophicephalus*	PP327214	*Proteus vulgaris* (KC210847)	98.30
NCIO28	*Menticirrhus ophicephalus*	PP327215	*Pseudoalteromonas* sp. (GU062519)	99.30
NCIO31	*Menticirrhus ophicephalus*	PP267455	*Psychrobacter immobilis* (NR_113805)	99.88
NCIO32	*Menticirrhus ophicephalus*	PP327270	*Shewanella* sp. (EU075118)	99.60
NCIO33	*Menticirrhus ophicephalus*	PP267456	*Psychrobacter cibarius* (NR_043057)	99.79

NCIO35	*Menticirrhus ophicephalus*	PP327366	*Shewanella* sp. (EU075116)	98.10
NCIS38	*Menticirrhus ophicephalus*	PP327367	*Aliivibrio finisterrensis* (EU541616)	94.10
NCIS39	*Menticirrhus ophicephalus*	PP267457	*Pseudoalteromonas elyakovii* (NR_028722)	99.25
NCIS40	*Menticirrhus ophicephalus*	PP328540	*Aliivibrio fischeri* (AY292949)	98.20
NCIS41	*Menticirrhus ophicephalus*	PP328541	*Aliivibrio fischeri* (AY292949)	97.10
NCIS42	*Menticirrhus ophicephalus*	PP328543	*Shewanella marinintestina* (AB081758)	100.0
NCIS43	*Menticirrhus ophicephalus*	PP328545	*Aliivibrio fischeri* (AY292949)	98.20
NCIA30	*Pinguipes chilensis*	PP328579	*Vibrio splendidus* (AJ874367)	100
NCIA31	*Pinguipes chilensis*	PP328590	*Vibrio* sp. (AF242274)	100
NCIA32	*Pinguipes chilensis*	PP328775	*Vibrio lentus* (AY292935)	100
NCIA33	*Pinguipes chilensis*	PP328776	*Vibrio* sp. (DQ328955)	100
NCIA34	*Pinguipes chilensis*	PP331442	*Vibrio* sp. (DQ480140)	94.00
NCIA35	*Pinguipes chilensis*	PP331448	*Vibrio* sp. (FJ457534)	100
NCIA36	*Pinguipes chilensis*	PP331491	*Vibrio* sp. (FR744824)	100
NCIA37	*Pinguipes chilensis*	PP331796	*Vibrio* sp. (FJ457352)	100
NCIS28	*Pinguipes chilensis*	PP267458	*Vibrio crassostreae* (NR_044078)	99.14
NCIA14	*Prolatilus jugularis*	PP267503	*Photobacterium carnosum* (NR_156814)	97.75
NCIA15	*Prolatilus jugularis*	PP331805	*Vibrio* sp. (AF242274)	99.20
NCIA16	*Prolatilus jugularis*	PP267504	*Vibrio alginolyticus* (NR_122050)	99.36
NCIA17	*Prolatilus jugularis*	PP267505	*Vibrio alginolyticus* (NR_122059)	99.36
NCIA18	*Prolatilus jugularis*	PP331845	*Shewanella woody* (JF412212)	97.10
NCIA19	*Prolatilus jugularis*	PP267506	*Photobacterium carnosum* (NR_156814)	98.18
NCIA20	*Prolatilus jugularis*	PP331856	*Vibrio kanaloae* (JN128262)	100
NCIS14	*Prolatilus jugularis*	PP332289	*Aliivibrio* sp. (EU862334)	100
NCIS14b	*Prolatilus jugularis*	PP332290	*Aliivibrio fischeri* (AY292920)	98.70
NCIS15b	*Prolatilus jugularis*		*Vibrio crassostreae* (NR_044078)	99.25
NCIS16	*Prolatilus jugularis*	PP332294	*Aliivibrio logei* (JF412238)	100
NCIS16b	*Prolatilus jugularis*	PP267551	*Vibrio chemaguriensis* (NR_179846)	99.18
NCIS17	*Prolatilus jugularis*	PP267552	*Photobacterium aquimaris* (NR_114269)	99.77
NCIS18	*Prolatilus jugularis*	PP332295	*Aliivibrio finisterrensis* (EU541614)	96.30
NCIS19	*Prolatilus jugularis*	PP332310	*Shewanella woodyi* (JF4122079)	98.10
NCIA12	*Genypterus chilensis*	PP333039	*Vibrio lentus* (AY292935)	100
NCIS13	*Genypterus chilensis*	PP332397	*Shewanella* sp. (FN295775)	99.30
NCIS15	*Genypterus chilensis*	PP270283	*Photobacterium sanguinican* (NR_146675)	99.79
